# Specialty preferences among medical students in Botswana

**DOI:** 10.1186/s13104-017-2523-y

**Published:** 2017-06-08

**Authors:** Ambrose Rukewe, W. A. Abebe, A. A. Fatiregun, M. Kgantshang

**Affiliations:** 1 Department of Anaesthesia, University of Botswana, Private Bag 00713, Gaborone, Botswana; 2 World Health Organisation, Akure Office, Akure, Ondo State Nigeria; 3 Department of Anaesthesia, Princess Marina Hospital, Gaborone, Botswana

**Keywords:** Specialty, Specialisation, Medical students, Botswana

## Abstract

**Background:**

With the establishment of a new medical college in Botswana to train generalist-doctors and specialists, we set out to explore the career preferences of medical students, factors that influence their choices and attitude to local postgraduate training.

**Methods:**

A descriptive cross-sectional questionnaire-based study was conducted among medical students in their third to fifth year, at the Faculty of Medicine, University of Botswana. The structured, self-administered questionnaires which were hand-delivered covered demographic characteristics of responders, career choices, preferred location of specialisation and factors that influenced the choices.

**Results:**

Of the 143 medical students approached, 116 (81.0%) returned completed questionnaires. Of the responders, 102 (87.9%) intend to pursue postgraduate specialisation against 2 (1.7%) who declined; 12 (10.3%) were undecided. The four most preferred specialties which constituted 68.1% were surgery (28.4%), paediatrics (19.0%), internal medicine (12.9%), obstetrics and gynaecology (7.2%). There was male preference for surgery (p = 0.04), while women were drawn more towards paediatrics and psychiatry (p = 0.04 and p = 0.01, respectively). Personal interest and aptitude was considered the most important factor among most responders (46.2%), followed by enjoyment of the posting (19.8%). A high proportion of responders 80 (69.0%) preferred to specialise abroad for better exposure/opportunities (48.3%), while for 15.5%, their preferred courses are not currently available locally.

**Conclusion:**

Our findings indicated that while four major specialties are preferred, significant gender differences exist with female students leaning towards non-surgical disciplines. Students prefer specialising abroad on the pretext that foreign centres offer better training opportunities, and many specialist programmes are unavailable locally.

**Electronic supplementary material:**

The online version of this article (doi:10.1186/s13104-017-2523-y) contains supplementary material, which is available to authorized users.

## Background

A country’s health care service is largely affected by the career choice and specialisations of its medical practitioners. This is even more important in many sub-Saharan African countries where acute and chronic shortages of skilled health personnel subsist [1, 2]. Although Botswana is not affected by such shortages, less than a quarter of the physician-population are citizens. This puts the country at risk if the largely expatriate doctors cannot be replaced on retirement or migrate to other countries [3, 4]. The establishment of University of Botswana Faculty of Medicine (UBFoM) in 2009, to train generalist-doctors and specialists was supposed to address the nation’s dependence on expatriates. The mission of the medical school was to produce highly trained generalist doctors who can function within primary care and the residency programme would recruit and train specialists that are easier to retain than foreign medical graduates. In 2011, the UBFoM started 4-year residency MMed programme in internal medicine, paediatrics, family medicine, emergency medicine, public health, pathology and anaesthesia, and graduated its first MBBS class in 2014.

Students are selected to enter the MBBS programme based on their A’ level results or results in the one year Pre-medical science or Bachelor of Science programmes. The 5-year UBFoM MBBS programme was divided into two phases; the pre-clinical phase lasting 2 years covered basic medical sciences, communication skills and introduction to community-based learning. The 3-year clinical phase consisted of five 8-week rotations in referral, district, primary hospitals and clinics in Gaborone and four community-based teaching platforms up to 1000 km from the Gaborone main campus. Each student during phase one and phase two programme is assigned a faculty member as academic advisor for building mentoring relationships. The 3rd year students were fifty-two (52) in number, 4th year fifty-four (54) and 5th year were thirty-seven (37), making a total of one hundred and forty-three (143).

To the best of our knowledge, no study has explored the career preferences of medical students in Botswana and their attitude to local postgraduate training. This study should provide data for professional bodies, training institution and Government to formulate policies to ensure a good mix of medical personnel of today and tomorrow. The objectives of this study were to examine specialisation preferences of medical students and factors influencing their choices as well as their preferred choice of location for specialisation.

## Methods

Following approval from the Institutional Ethics Committee, a descriptive cross-sectional study was conducted among medical students at UBFoM. A total of 143 students were approached. Our cohort comprised the medical students in the 3rd, 4th and 5th years. Written informed consent was obtained from each participant. Information was obtained using a structured, self-administered questionnaire (Additional file 1), which was hand-delivered. The questionnaire focused on gender, age, marital status, choice of specialty and timing of decision, the preferred location of training (UBFoM or abroad) and reason for choosing a particular location.

The questionnaire was pretested among selected preclinical students and modified before administration. Each respondent was allowed to pick only one specialty, the most important factor that informed the career choice and the preferred location of specialisation. The data collected were analysed using descriptive statistics by the statistical package for the social sciences (SPSS for windows 21.0, SPSS Inc., Chicago, IL, USA). Chi square test was used to evaluate gender differences of career choices and preferred location of specialisation. A *P* value of <0.05 was considered statistically significant.

## Results

Of the 143 clinical students, 116 (81.0%) returned completed questionnaires; over half 60 (51.7%) were males. Of our cohort, 45 (38.8%) were 3rd year students, 40 (34.5%) students were 4th year and 31 (26.7%) were in their 5th year. The mean age of the respondents was 23.5 (± 1.7) years, a high proportion (99.1%) were single. Of the responders, 102 (87.9%) intend to specialise against 2 (1.7%) who have no plans to pursue a postgraduate career; 12 (10.3%) were undecided.

### Timing of decision to specialize

Most of the responders decided about their career during the clinical years 74/116 (63.8%); a high proportion (43.5%) in the 3rd year. The remaining 42 (36.2%) students were either undecided or made their choices outside this period.

### Specialty choices and characteristics

**Fig. 1:**
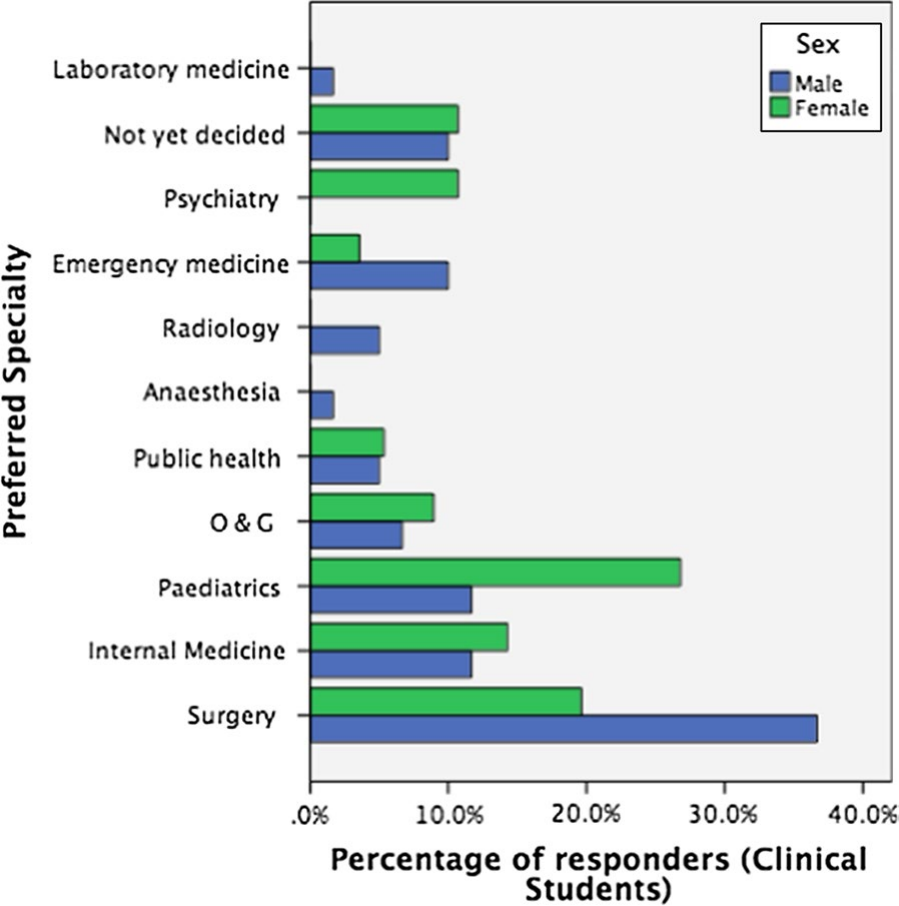
Specialty preferences by gender among responders

The four most preferred specialties which constituted 79/116 (68.1%) were surgery (28.4%), paediatrics (19.0%), internal medicine (12.9%), obstetrics and gynaecology (7.8%). Figure 1 shows the medical students’ preferred specialties by gender. The choice along gender lines showed male preference for surgery (p = 0.04), while women were more drawn towards paediatrics and psychiatry (p = 0.04 and p = 0.01, respectively). One-tenth of the responders were undecided. No student picked any of the basic medical sciences or family medicine as an option. Disciplines such as radiology, anaesthesia and laboratory medicine were under-subscribed, and attracted men exclusively.

### Reasons for specialty choice

**Fig. 2:**
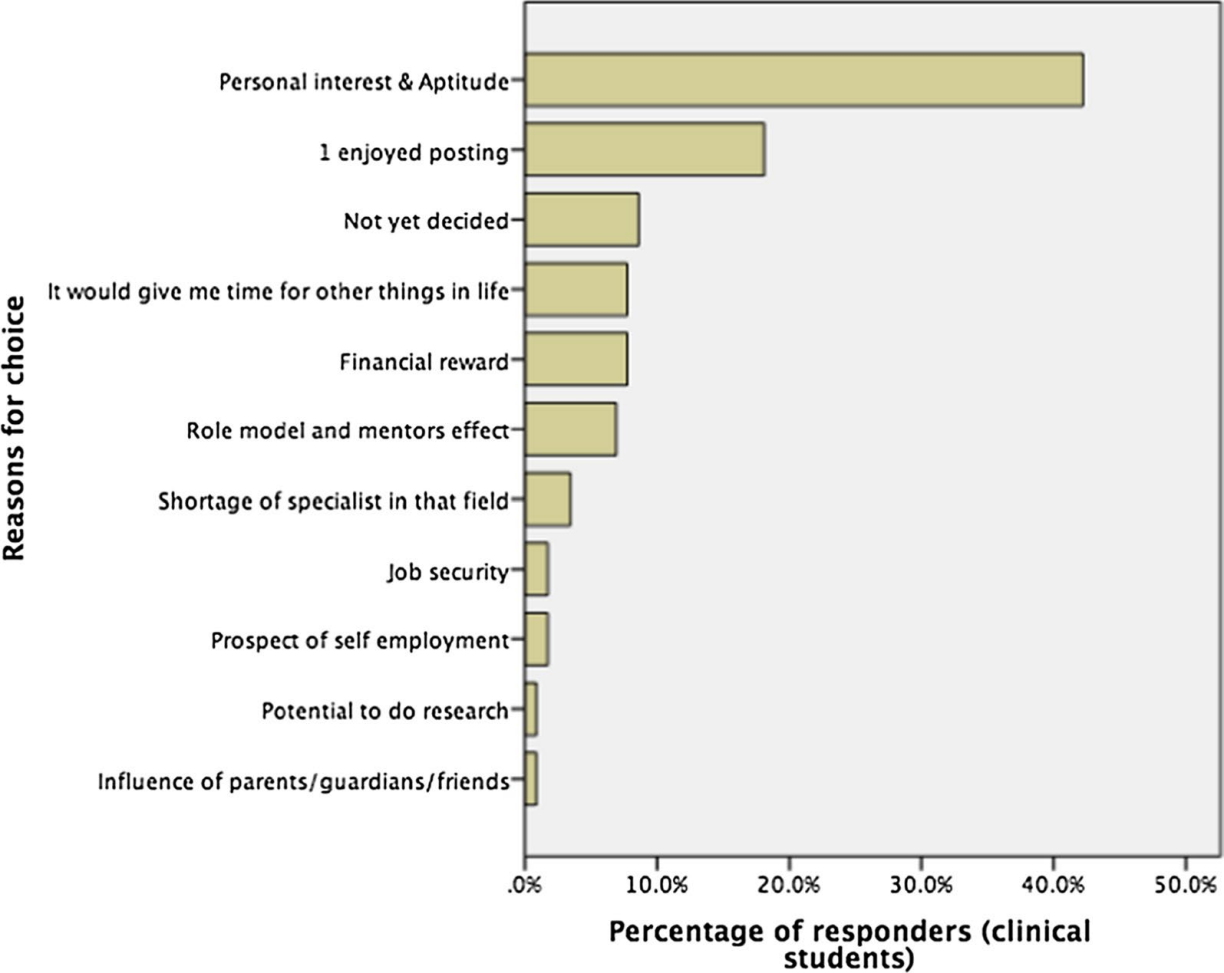
Factors influencing choice of specialty among responders

Personal interest and aptitude was considered the most important reason by most responders 49/106 (46.2%) who intended to specialise followed by enjoyment of the posting 21 (19.8%). According to Fig. 2, other contributory factors are “easy working hours” (8.5%), financial reward (7.8%) and effect of role models/mentors (7.6%).

### Preferred location to specialise

A high proportion of responders 80/116 (69.0%) preferred to go abroad to specialise; 25 (21.6%) chose University of Botswana (UB) while 11 (9.5%) are indifferent (Table 1). The predominant reason for the choice of abroad is better exposure/opportunities (48.3%). According to 15.5% respondents, their preferred courses are not currently available locally. Altruism and convenience rank high among respondents (22.4%) who opt to specialise in UBFoM (Table 2). Although our data showed that female students were more inclined to training abroad than their male counterparts, there was no significant association (P = 0.788).

**Table 1:** Preferred location of specialisation by gender

	Male, n (%)	Female, n (%)
Abroad	40 (66.7)	40 (71.4)
University of Botswana (UB)	14 (23.3)	11 (19.6)
Indifferent	6 (10.0)	5 (8.9)
Total	60 (100.0)	56 (100.0)

**Table 2:** Factors influencing choice of training abroad

	n (%)
Better exposure and opportunities outside UB	56 (48.3)
Convenience and altruism	26 (22.4)
Specialty not currently offered in UB	18 (15.5)
Others	12 (10.3)
Financial reward	3 (2.6)
Short learning period	1 (0.9)
Total	116 (100.0)

## Discussion

In this present study, four major disciplines (surgery, paediatrics, internal medicine, and obstetrics & gynaecology) are the most desirable specialties among medical students of the University of Botswana. This is similar to reports among medical students in the Gambia, Greece, Kenya and Jordan [5–8]. This tendency might be a direct result of the fact that medical students spend ample time in these specialties to the detriment of others and therefore, find them more appealing, a situation that has been described as medical socialisation [9]. Our male respondents were more inclined towards surgery and emergency medicine while there was significant association between women and non-surgical specialties (paediatrics and internal medicine). One Kenyan survey in 2010 reported the same preference along gender lines [1].

It is noteworthy that a high proportion (87.9%) of our students wishes to pursue postgraduate medical studies, a figure which is much <97.6% reported among Greek medical students [10]. This strong tendency toward specialisation can be said to portend good for the future of medical practice in Botswana given that the more specialists are available the better the potential to reach and maintain the highest attainable level of health of the citizenry [11]. The low level of interest among our respondents in family medicine, anaesthesia, public health, laboratory medicine and basic medical sciences is consistent with other studies [5, 6, 12–14]. The effect of this lack of interest in these specialties is that there will be a shortfall in postgraduate entrants in these fields and on the long run, shortage of particular specialists. The UBFoM can ill afford this as it has the mandate to train generalist doctors and specialists in order the reverse the nation’s reliance on expatriate medical workforce [3, 4, 15]. This calls for strategies to attract students to less glamorous disciplines because the country’s health needs are better served with adequate and appropriate health resource. This is imperative to maintain her better health indices compared with neighbouring South African countries [16].

The most important reason for choosing a specialty was personal interest and aptitude, similar to the findings in other studies [12, 14]. This is at variance with other studies, which reported extrinsic factors such as remuneration, prospect of self-employment, influence of mentors and easy working hours as determinants [8, 13, 17]. A high proportion of students mentioned, “enjoyment of posting” as an influence on their career choice. This strengthens the notion that the amount of time given to poorly selected specialties in the medical curriculum does not allow students to develop interest or see potential in them. In the United Kingdom, an undergraduate curriculum reform was carried out that allowed additional 4-weeks attachment in general practice with a more positive attitude towards the specialisation [14, 18]. The UBFoM can take a cue from this and allot more time to less fancied specialties in future undergraduate curriculum review. Other strategies such as selection of students from rural backgrounds and provision of incentives for graduates to pursue career in family medicine could generate interest in the specialty. Our students could benefit from career counseling in line with the Gambian study on this subject [5].

Our results indicated a high proportion of students (69.0%) preferred specialising abroad, similar to the Greek and Arscott-Mills et al’s studies earlier alluded to [10, 15]. The students based their choice on better exposure/opportunities coupled with the fact that their preferred specialties are not available locally. The affected disciplines: surgery, obstetrics and gynaecology, radiology and psychiatry polled 49.0% in favourability rating if one excluded those undecided. The Greek and Botswana’s situations are two sides of a coin: here, some specialties do not have postgraduate training programme and in the other, there are more candidates for the available positions so students have long waiting periods before entry into preferred specialties. The burden is on UBFoM to fast-track faculty development and capacity building to institute specialist training in the affected disciplines. It has been adduced that the absence of specialist training programmes contribute to the difficulty in retaining citizen-doctors and it could be the impetus to attract foreign-trained Batswana physicians to return home to practice [4].

An important limitation of this study is the fact that we have measured future career choices at a set time during medical school which may change in later years or during internship period, when the newly-qualified doctors rotate through surgery, medicine, paediatrics and obstetrics and gynaecology. The very fact that 12 (10.3%) responders were undecided at this point in time should put this into perspective. It should be borne in mind that we have restricted our respondents to only one option for career and related factors, people tend to have more than one option in real-life situations. We wish to recommend a prospective cohort study of these medical students after internship knowing that their exposure to certain disciplines could impact on earlier decisions and refine their choice of specialties.

## Conclusions

In conclusion, a high proportion (87.9%) of our students intends to pursue postgraduate specialisation in one of the four major specialties (surgery, paediatrics, internal medicine and obstetrics and gynaecology). However, significant gender differences exist with female students leaning towards non-surgical disciplines. Students prefer specialising abroad on the pretext that foreign centres offer better training opportunities, and many specialist programmes are unavailable locally. This study has highlighted the career preferences and attitude of medical students in Botswana to local postgraduate training and should provide data for stakeholders towards actualising the mission of the medical school.

## Additional file

**Additional file 1: Appendix S1. ** Questionnaire. The survey questions presented to the medical students for their response.

## Abbreviations

UBFoM: University of Botswana Faculty of Medicine
UB: University of Botswana
