# Supplementary material for: Specialty preferences among medical students in Botswana
Source: BMC Res Notes. 2017 Jun 8;10:195. doi: 10.1186/s13104-017-2523-y (PMC5465444; doi:10.1186/s13104-017-2523-y)
Supplement: Supplementary file 1 — Additional file 1: Appendix S1. Questionnaire. The survey questions presented to the medical students for their response. [file 13104_2017_2523_MOESM1_ESM.docx]

**Appendix S1: Questionnaire**

**Re: Specialty preferences among medical students in Botswana**

This study has been approved by UB Office of Research & Development, IRB Research approval number: UBR/RES/IRB/1656. Principal Investigator: Dr. Ambrose Rukewe.

The goal of this study is to investigate the specialty preferences of medical students as well as the factors that influence their choices. We hope that this study would generate data that will be useful for long term planning of the medical workforce in the development of health care services in Botswana. It should take less than 5 minutes to complete the questionnaire. Your response to these questions would serve the purpose of this study and will be confidential. Thank you for your cooperation.

1. Serial no…………**(Omit question 1 only)**
2. Age ………………yrs
3. Sex (a) Male (b) Female
4. Marital status (a) single (b) married (c) divorced (d) widowed
5. Status (a) 3^rd^ Yr MS (b) 4^th^ Yr MS (c) 5^th^ Yr MS
6. Do you intend to specialise after MBBS degree? 1. Yes 2. No 3. Not yet decided
7. If yes, what is your preferred specialty? (a) Surgery (b) Internal medicine (c) Paediatrics (d) O & G (e) Public health (f) Family medicine (g) Basic medical sciences e.g. Anatomy, Physiology, etc (h) Anaesthesia (i) Radiology (j) Ophthalmology (k) ENT (l) Emergency Medicine (m) Psychiatry (n) orthopedic

(o) others…………………………………………………**(Fill in choice, if not listed)**

1. When did you make that decision?...........................**(state year of study)**
2. What factor affected your choice **PICK THE MOST IMPORTANT** (a) Personal interest & Aptitude (b) Financial reward (c) Prestige (d) Prospect of self-employment (e) Job security (f) I enjoyed the posting (g) It would give me time for other things in life (h) Role model effect (i) Potential to do research (j) Shortage of specialist in that field (k) Influence of consultants/mentors (l) Influence of parents/guardian/friends (m) **if option is not listed above** …………………………………………………………...
3. Where would you like to specialise? 1. UB 2. Abroad
4. State reason for your choice of location………………………………………………...
